# Genome Sequence of *Serratia plymuthica* Strain S13, an Endophyte with Germination- and Plant-Growth-Promoting Activity from the Flower of Styrian Oil Pumpkin

**DOI:** 10.1128/genomeA.00594-13

**Published:** 2013-08-08

**Authors:** Henry Müller, Michael Fürnkranz, Martin Grube, Gabriele Berg

**Affiliations:** Institute of Environmental Biotechnology, Graz University of Technology, Graz, Austriaa; Institute of Plant Sciences, Karl-Franzens-University, Graz, Austriab

## Abstract

The bacterium *Serratia plymuthica* strain S13 was demonstrated to colonize various plant-associated microhabitats and to suppress damping-off diseases. The completed genome sequence has a size of 5.5 Mb, containing 4,957 putative protein-encoding regions, and will be used to identify genetic determinants enabling the bacterium to escort a plant’s entire life cycle.

## GENOME ANNOUNCEMENT

Members of the enterobacterial genus *Serratia* have been reported to be associated with many plants, where they perform beneficial functions by suppressing phytopathogens and promoting growth (1). The strain *Serratia plymuthica* S13 was isolated from the anthosphere of Styrian oil pumpkin (*Cucurbita pepo* L. subsp. *pepo* var. *styriaca*) but was also shown to colonize the rhizosphere, endosphere, and seeds of the same plant species (2). S13 was selected as a strong antagonist toward *Didymella bryoniae*, the causal agent of black rot in pumpkins. When applied as a seed treatment, *S. plymuthica* enhanced the germination rate and controlled damping-off diseases under field conditions with efficiency comparable to that of chemical fungicides containing copper, metalaxyl-M, or fludioxonil (3). The genome was sequenced to reveal the genetic predisposition that facilitates versatile microniche colonization and seed-protecting capacity.

The *S. plymuthica* S13 genome sequence was obtained using the Roche/454 GS-FLX Titanium sequencing platform. A draft assembly based on 339,698 reads of a standard shotgun library and 427,170 reads of an 8-kbp paired-end library (LGC Genomics, Berlin, Germany) with a total of 191.6 Mb (35-fold coverage) was generated with Newbler assembler (software release 2.6) (Roche Diagnostics GmbH, Mannheim, Germany). This assembly consisted of 34 contigs, 27 of which could be joined into a single circular scaffold. Gaps resulting from repetitive sequences were resolved by *in silico* gap filling; remaining gaps were closed by PCR followed by Sanger sequencing, yielding a final genome of 5,467,306 bp.

Genes were identified with the software tool Prodigal 1/1/20 (4). Functional annotation of the predicted genes was performed by use of the GenDB system 2.2 (5) and JCoast 1.7 (6), which provided annotations with respect to clusters of orthologous groups (COG) (7), Pfam (8), and gene ontology (GO) (9). The final genome includes 5.4 Mb, with a GC content of 56.19%. The number of putative genes totals 4,957, of which 4,866 are protein coding. There are seven instances of the 5S-23S-to-16S rRNA cluster and 84 tRNAs.

We identified putative genetic elements enabling beneficial interaction with the plant as well as antagonism toward fungal pathogens. Bacterium-plant interaction was assumed to be conferred by genes involved in plant growth hormone synthesis/signaling (auxin, salicylic acid, and butandiol) and root colonization (type I secretion system, adhesin, and hemagglutinin). Other genes encoding fungal cell-wall-degrading enzymes (chitinases and β-1,3 glucanases) and nonribosomal peptides (syringomycin and siderophores) are probably involved in the suppression of fungi.

### Nucleotide sequence accession number.

The *Serratia plymuthica* S13 genome sequence and annotation data have been deposited in GenBank under the accession number CP006566.
